# Announcement

**Published:** 2015-08-14

## Community Preventive Services Task Force Issues *2014–2015 Annual Report to Congress*

The Community Preventive Services Task Force recently posted a new report on its website: *2014–2015 Annual Report to Congress, Federal Agencies, and Prevention Stakeholders.* This report includes a special update on task force recommendations to prevent cancers and is available at http://www.thecommunityguide.org/annualreport.

Established in 1996 by the U.S. Department of Health and Human Services, the task force is an independent, nonfederal, unpaid panel of public health and prevention experts whose members are appointed by the Director of CDC. The task force provides information for decision makers on programs, services, and policies aimed at improving population health. Although CDC provides administrative, research, and technical support for the task force, the recommendations developed are those of the task force and do not undergo review or approval by CDC.

